# List of Diseases at the Bath City Dispensary, from February 1, to May 1, 1802

**Published:** 1802-06

**Authors:** 


*List of Diseases at the Bath City Dispensary, from February* 1, *to May* 1, 1802.

| | | | |
|---|---|---|---|
| Angina | 8 | Pleuritis | 9 |
| Arthritis | 1 | Pneumonia | 1 |
| Asthma | 11 | Phthisical | 27 |
| Abscess | 3 | Phthisis Scrophulosa | 1 |
| Anasarca | 7 | Pertussis | 8 |
| Ambustiones | 3 | Peripneumonia Notha | 1 |
| Bowel Complaints | 13 | Pulmonary Complaints | 11 |
| Catarrh | 20 | Paralysis | 1 |
| Cynanche Parotidœa | 1 | Phelgmatia Dolens | 2 |
| Cholera | 2 | Pleurodyne | 2 |
| Cholic | 2 | Psora | 6 |
| Chlorosis | 5 | Prolapsus Uteri | 1 |
| Diarrhœa | 4 | Rubeola | 2 |
| Dysuria | 1 | Rheumatism | 25 |
| Diabetes Mellitus | 1 | Rachitis | 1 |
| Erysipelas | 3 | Synochus Biliosa | 23 |
| Empyema Psoadicum | 1 | ——— Pleuritica | 2 |
| Epistaxis | 1 | ——— Enteritica | 1 |
| Eruptiones | 3 | ——— Dysenterica | 3 |
| Fever | 17 | ——— Cholerica | 2 |
| Fistula in Ano | 1 | Scarlatina | 2 |
| Fractured Rib | 1 | Syphilis | 14 |
| Gravel | 2 | Suppuration | 2 |
| Gastrodynia | 9 | Shingles | 1 |
| Hydrocephalus Internus | 1 | Scrophula | 5 |
| Hæmoptysis | 3 | Scirrhus | 2 |
| Hæmorrhagia | 2 | Strain | 7 |
| Hernia | 1 | Strictures in Urethra | 7 |
| Hernia Humoralis | 1 | Typhus | 3 |
| Herpes | 3 | Tussis | 13 |
| Inflammation | 2 | —— cum Dyspnœa | 9 |
| Iliac Passion | 1 | Tinea Capitis | 4 |
| Infantile Complaints | 4 | Tumor | 4 |
| Icterus | 1 | Ulcus | 4 |
| Incurvated Spine | 1 | Ulcerated Legs | 10 |
| Lepra | 3 | Variola | 1 |
| Leucorrhœa | 3 | Vermes | 5 |
| Menorrhagia | 5 | | |
| Nephritic Complaints | 3 | Total | 378 |
| Ophthalmia | 9 | | |

